# Suppression of transgenerational lipid provisioning inhibits desiccation resistance, but not diapause, in the vector mosquito, Aedes albopictus

**DOI:** 10.21203/rs.3.rs-8302219/v1

**Published:** 2026-01-30

**Authors:** Mara Heilig, Marten J. Edwards, Peter A. Armbruster

**Affiliations:** Georgetown University; Muhlenberg College; Georgetown University

**Keywords:** transgenerational plasticity, overwinter biology, lipid energetics, diapause, Aedes albopictus

## Abstract

Transgenerational signaling allows many organisms to anticipate seasonal variation. Diapause, a hormonally-programmed period of developmental arrest, is a widespread anticipatory seasonal response that is often mediated by transgenerational signaling. Despite the well-established adaptive significance of diapause, the molecular and physiological basis of transgenerational diapause signaling remains largely unresolved. Since lipid accumulation is a hallmark of diapause preparation, we hypothesized that increased lipid provisioning and accumulation mediate transgenerational diapause signaling. To test this hypothesis, we suppressed transcripts of two lipid metabolism genes using RNA-interference in the maternal generation of the vector mosquito, *Aedes albopictus*. We show that a reduction in maternal *lipid storage droplet 2* (*lsd2)* transcript abundance, but not *diacyl-glycerol O-acyltransferase 1* (*dgat1*) transcript abundance, reduces egg triglyceride levels. Suppression of *lsd2* in adult females also leads to increased egg desiccation, inhibits the starvation tolerance of larvae, and decreases egg overwinter survival. However, knockdown of *lsd2* does not affect diapause incidence, the timing of diapause termination, and post-diapause development. Together, our results indicate that transgenerational lipid provisioning affects diapause-associated overwintering fitness traits, but not the regulation of entry into diapause or termination of diapause.

## INTRODUCTION

Phenotypic plasticity enables individuals to express distinct morphological, physiological, and behavioral phenotypes depending on environmental conditions [1, 2]. For example, to persist in seasonally fluctuating environments, many organisms exhibit adaptive forms of anticipatory phenotypic plasticity that allow them to initiate physiological and/or developmental alterations in advance of seasonally changing environmental conditions [3, 4]. Moreover, anticipatory plasticity can occur across generations, a process known as transgenerational plasticity [5, 6]. Determining the molecular basis of transgenerational plasticity has been a longstanding question in the fields of organismal and evolutionary biology [7].

Diapause is a widespread form of anticipatory phenotypic plasticity characterized by a halt in development, decreased metabolism and increased stress tolerance [3]. Diapause often involves transgenerational signaling to link three distinct eco-physiological phases: pre-diapause, diapause and post-diapause [8, 9]. Diapause induction is the first stage of pre-diapause, when a “token” environmental cue, often daylength (photoperiod), is perceived prior to the onset of unfavorable conditions. Next, the organism prepares for diapause by making physiological, behavioral and/or metabolic adjustments that prepare it, or its offspring, for a prolonged developmental arrest. This preparatory phase often occurs across generations among organisms that undergo a maternally-mediated photoperiodic diapause response. During diapause, metabolism is suppressed and developmental arrest is maintained for a genetically determined period of time, even if conditions become favorable. Finally, diapause termination leads to the post-diapause phase, when the organism becomes receptive to environmental conditions that promote growth and development.

The specific molecular signals that are transmitted between generations to regulate maternally mediated diapause are generally poorly understood [5]. One notable exception involves the induction of maternal diapause in the silk moth, *Bombyx mori*. When the maternal generation is exposed to diapause-inducing conditions, females release a neuropeptide from the suboesophageal ganglia known as diapause-hormone [10–12]. Diapause-hormone targets the ovarioles of adult females and leads to the production of eggs with increased levels of sorbitol and glycerol. Embryos developing from these eggs enter diapause at the G2 stage [13], and remain in a state of arrested development until sorbitol has been degraded to low concentrations [11]. Thus, diapause hormone and sorbitol are key components of diapause induction and termination in *B. mori.* However, neither has been implicated as a transgenerational signal for diapause induction or termination in non-lepidopteran. Nevertheless, the work in *B. mori* establishes the principle that both hormones and nutritional provisioning can be components of diapause regulation, but that the specific molecular signals might differ between species and life stages.

Nutritional signaling, involving both maternal nutrient provisioning and offspring nutrient sensing, is a compelling mechanism for transgenerational diapause regulation [14–16]. Most insects do not feed throughout diapause, making increased nutrient accumulation and storage critical elements of the diapause phenotype [14, 16, 17]. This is because individuals need stored energy to repair damaged tissue and to fuel survival throughout diapause and diapause termination. Since lipids are the primary source of metabolic fuel stored during diapause, and triglycerides are the main form of energetic lipid [16, 17], there is particular interest in understanding the role of triglycerides during diapause. Notably, studies linking diapause-associated lipid accumulation with insulin signaling and the target of rapamycin (TOR) pathway provide further indirect evidence that nutrient sensing and signaling may regulate diapause [18–20]. However, studies that have directly manipulated lipid quantities to determine their functional role in diapause remain limited and have yet to establish that lipid sensing regulates diapause [21, 22]. Additionally, no studies have manipulated lipid provisioning across generations to test for an effect on diapause initiation or termination.

The vector mosquito, *Aedes albopictus*, is a useful model for studying transgenerational diapause regulation [23]. Female pupae and adults from temperate populations perceive the short daylength of late summer/early autumn and produce offspring that complete embryonic development but enter diapause as pharate larvae within the chorion of the egg [24, 25]. Although there is strong evidence that the proximal signal for developmental arrest in *Ae. albopictus* is low concentrations of juvenile hormone (JH III) [26, 27], the mechanisms by which that signal is transferred across generations remain unknown. However, multiple studies indicate that nutritional status and lipid metabolism are important facets of diapause preparation and diapause maintenance in *Ae. albopictus* [28–30]. For example, relative to non-diapause eggs, diapause eggs have greater overall lipid abundance [30], higher quantities of diglycerides and triglycerides [28], and altered metabolite profiles indicative of increased lipid storage and decreased lipid catabolism [28]. Additionally, metabolic genes involved in gluconeogenesis and lipid metabolism are differentially expressed throughout diapause preparation in both adult females exposed to diapause-inducing short-day photoperiod, and in diapause destined embryos [25, 30, 31]. These gene expression changes generally correspond with increased nutrient synthesis and storage in diapause relative to non-diapause eggs.

Directly altering nutrient levels through genetic manipulations is a powerful approach to evaluate the role of specific energetic reserves during diapause. For example, this approach can isolate the role of individual nutrients from confounding factors associated with dietary manipulation which may simultaneously alter multiple nutritional compounds and metabolic processes [14]. Herein, we altered transcript abundance of two genes with functions in lipid metabolism to evaluate transgenerational diapause signaling and diapause-associated performance traits. The first gene, *lipid storage droplet 2* (*lsd2)*, is expressed in the fat body and germ line of female *Drosophila melanogaster*. The protein localizes to the surface of lipid droplets [18, 32, 33], and is required for proper storage and deposition of neutral lipids during mid-oogenesis, ultimately affecting lipid deposition in eggs [32, 34, 35]. In *Ae. albopictus*, *lsd2* transcripts are ~ 2.2 fold more abundant in six-day old diapause-destined embryos, making it a strong candidate for contributing to diapause-related lipid storage [25]. The second gene, *diacyl-glycerol O-acyltransferase 1* (*dgat1*), catalyzes a rate-limiting step in the conversion of diglycerides to triglycerides. In *D. melanogaster*, *midway (mdy)* encodes a DGAT enzyme that is enriched in nurse cells and important for egg chamber development as well as the formation of lipid droplets (Harris et al. 2011) [36]. In this study, we used RNA-interference (RNAi) targeting *lsd2* and *dgat1* in the maternal generation of *Ae. albopictus* to determine if these genes contribute to the transgenerational provisioning of triglycerides and thereby regulate diapause incidence. We also determined the impact of *lsd2* and *dgat1* knockdown on the timing of diapause termination, egg desiccation resistance, overwinter survival and post-hatch larval starvation tolerance.

## RESULTS

### qRT-PCR to Assess RNAi Efficiency

**Figure 1:**
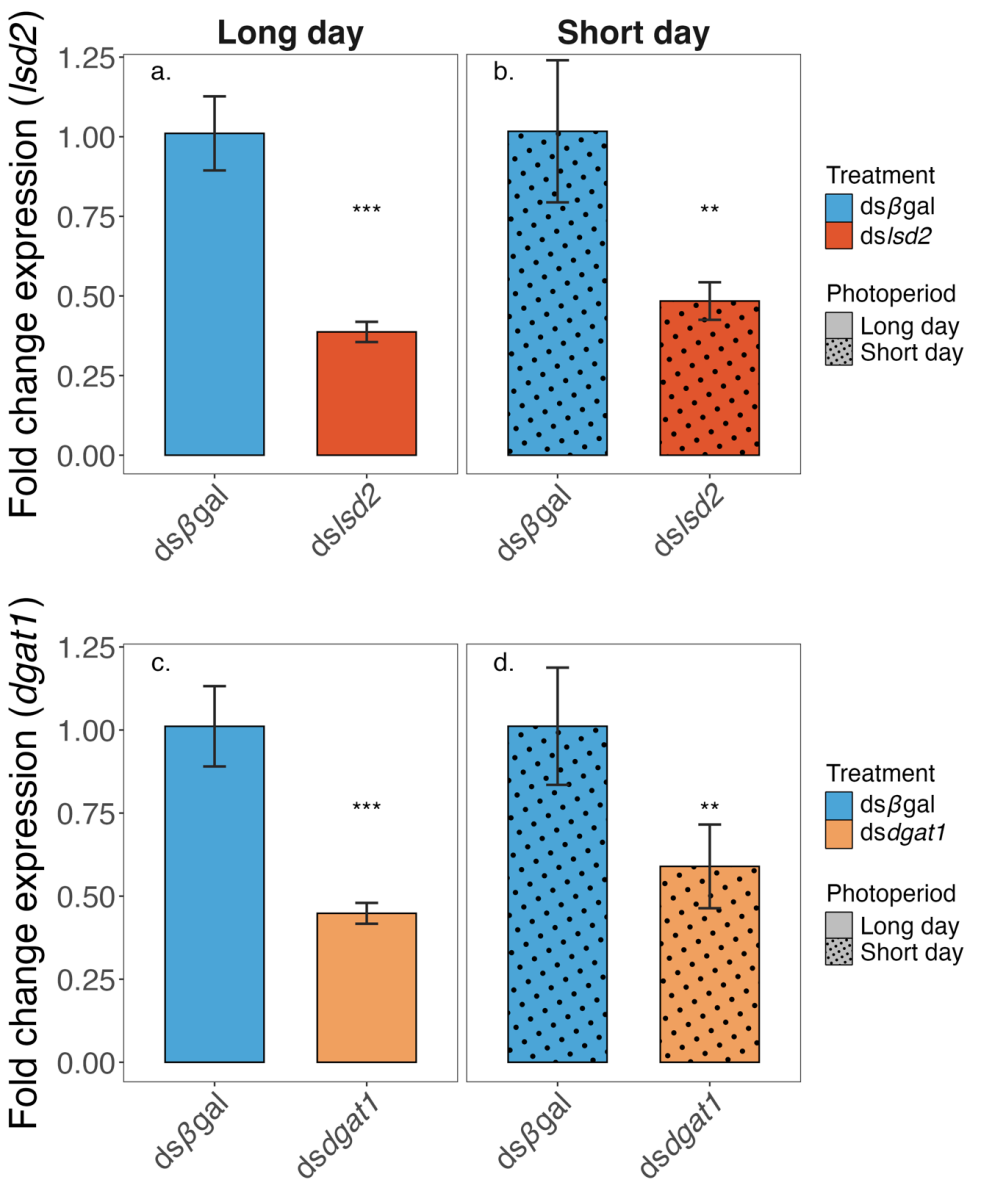
Injection of ds*lsd2* and ds*dgat1* reduce the transcript abundance of *lsd2* and *dgat1* in 3-day post-blood meal *Ae. albopictus* females under both long-day and short-day photoperiods. The relative fold change of each target gene is compared to females injected with ds*bgal*. Transcript levels were measured by qRT-PCR using *Ae. albopictus* ribosomal protein S6 (*AalRpS6*) as the reference gene. Top panels show: (a) Long-day, *lsd2* RNAi, (b) Short-day, *lsd2* RNAi; bottom panels show (c) Long-day, *dgat1* RNAi, (d) Short-day, *dgat1* RNAi. Data are shown as the mean ± 2 SE; ** indicates *P* < 0.01, *** indicates *P* < 0.001.

RNAi against *lsd2* significantly reduced *lsd2* mRNA levels under both long-day (LD) (Fig. 1a) (*Wilcoxon rank sum test, W* = 64, *P* < 0.001; 62% reduction) and short-day (SD) (Fig. 1b) (Student’s *t*-test, *t* = 4.62, *P* < 0.01; 54% reduction) conditions. Similarly, RNAi against *dgat1* significantly reduced *dgat1* mRNA levels under both LD (Fig. 1c) (Student’s *t*-test, *t* = 3.89, *P* < 0.01; 41% reduction) and SD (Fig. 1d) (*Wilcoxon rank sum test, W* = 64, *P* < 0.001; 56% reduction) conditions.

### Triglyceride Quantification

**Figure 2:**
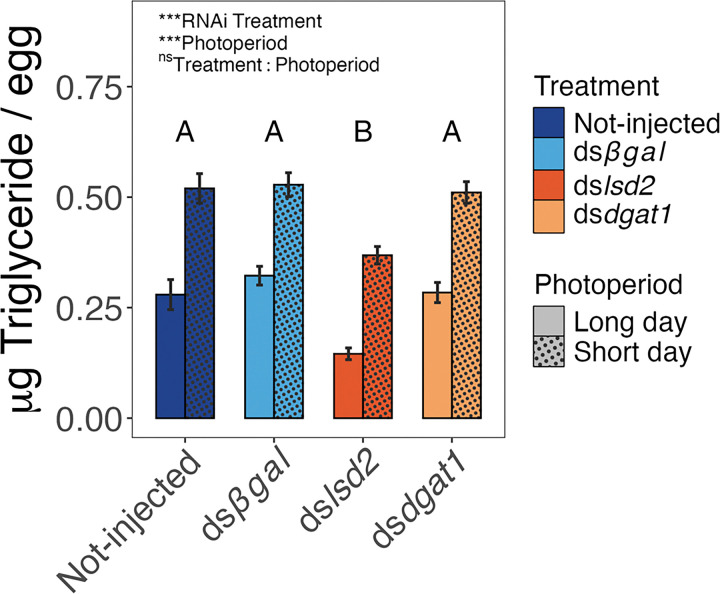
Injection of ds*lsd2* but not ds*dgat1* reduce egg triglyceride levels. Data show mean ± 2 SE μg triglyceride per egg produced by females injected with ds*lsd2*, ds*dgat1*, ds*bgal,* or not-injected under long-day or short-day conditions. Legend in the upper left indicates effect of RNAi treatment, Photoperiod, and RNAi treatment-by-photoperiod interaction (Two-way ANOVA); *** = *P* < 0.001, ns = *P* > 0.05. Letters indicate significant pairwise differences among RNAi treatment groups (averaged across photoperiods; Tukey HSD post-hoc test).

Egg triglyceride levels were impacted by both RNAi treatment (ANOVA, *F*_3,40_ = 72.70, *P* < 0.001) and photoperiod (ANOVA, *F*_1,40_ = 626.88, *P* < 0.001), but not by an RNAi treatment-by-photoperiod interaction (*F*_3,40_ = 0.627, *P* = 0.602) (Fig. 2). *Post hoc* Tukey’s HSD test revealed eggs produced by ds*lsd2* injected mothers under LD and SD photoperiods contained less triglyceride than eggs produced by ds*dgat1*, non-injected and ds*βgal* injected mothers under the same photoperiod (all *P*-values < 0.001). Under SD conditions, RNAi against *lsd2* reduced egg triglycerides by 27.8–30.1% compared to all other SD treatments. Under LD conditions, RNAi against *lsd2* reduced egg triglycerides 47.7–54.7% compared to all other LD treatments. Triglyceride levels of the ds*dgat1*, non-injected and ds*βgal* treatments within photoperiods did not differ (all *P-*values > 0.05).

### Starvation Tolerance

**Figure 3:**
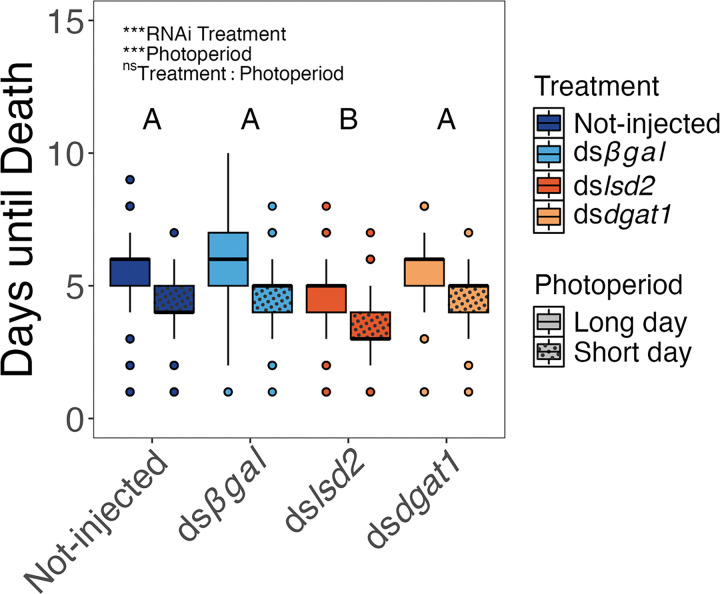
Injection of ds*lsd2* but not ds*dgat1* reduce starvation tolerance (= Days until Death) of 1^st^ instar larvae under both long-day and short-day conditions. Box plots show the distribution of days until death for larvae from different maternal RNAi treatments, hatched under long-day or short-day photoperiods. Box boundaries represent the 25th and 75th percentiles, the horizontal line within each box represents the median, and whiskers extend to 1.5 times the interquartile range. Points beyond whiskers represent outliers. Statistical results from GLM shown at top. Letters indicate significant pairwise differences among RNAi treatment groups (averaged across photoperiods; Tukey HSD post-hoc test).

Larval starvation tolerance (days until death) was impacted by RNAi treatment (χ^2^_3_ = 80.70, *P* < 0.001) and photoperiod (χ^2^_1_ = 115.95, *P* < 0.001), but not by an RNAi treatment-by-photoperiod interaction (χ^2^_3_ = 2.35, *P* = 0.504) (Fig. 3). Pairwise comparisons revealed that larvae produced by mothers injected with ds*lsd2* died significantly earlier than all other treatments (all *P*-values < 0.01). No other treatments showed significant pairwise differences (all *P*-values > 0.05).

### Diapause Incidence

**Figure 4:**
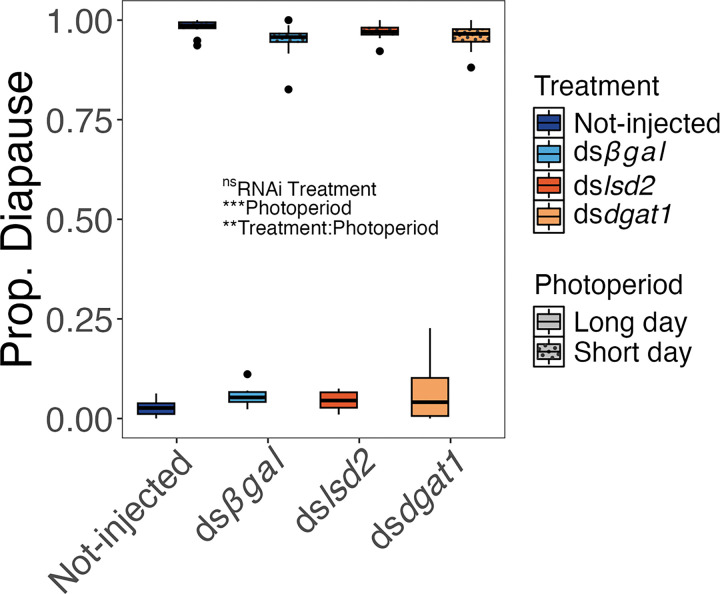
Maternal RNAi treatment does not impact diapause incidence under either long-day or short-day conditions. Each dot represents the proportion of eggs in diapause that were collected from a single cage. Symbols and conventions as in Fig. 3.

The proportion of eggs that entered diapause was between 3–7% across all treatments under LD conditions and between 94–98% across all treatments under SD conditions (Fig. 4). The proportion of eggs entering diapause was significantly affected by photoperiod (*F*_1,74_ = 2410.4, *P* < 0.001) and the interaction between photoperiod and RNAi treatment (*F*_3,71_ = 4.22, *P* = 0.008). However, diapause incidence was not impacted by RNAi treatment alone (*F*_3,74_ = 0.34, *P* = 0.795) (Fig. 4). All pairwise LD vs SD comparisons were significantly different across and within treatments (all *P* < 0.001), while no pairwise differences within photoperiod, across treatments were different (all *P* > 0.05).

### Desiccation Resistance

**Figure 5:**
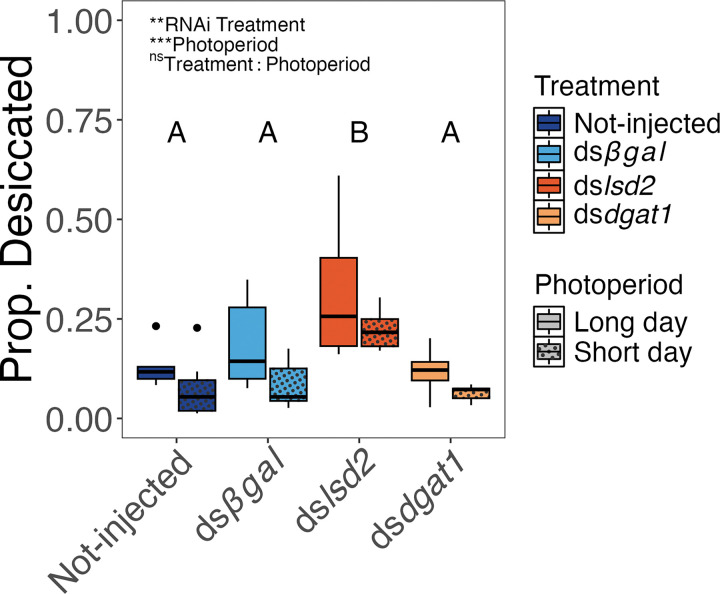
Injection of ds*lsd2* but not ds*dgat1* increases proportion of desiccated eggs. Box plots show the distribution of desiccation rates for eggs following different maternal RNAi treatments under both long-day and short-day photoperiods. Symbols and conventions as in Fig. 3.

The susceptibility of eggs to desiccation was significantly impacted by RNAi treatment (*F*_3,55_ = 18.34, *P* < 0.001) and photoperiod (*F*_1,55_ = 11.23, df = 1, *P* = 0.001), but not by an RNAi treatment-by-photoperiod interaction (*F*_3,52_ = 0.43, *P* = 0.732) (Fig. 5). Pairwise comparisons revealed eggs produced by d*slsd2* injected mothers were more prone to desiccation than eggs from any other treatment groups (averaged across photoperiods; Tukey HSD post-hoc test) (all *P* < 0.001).

### Diapause Duration

**Figure 6:**
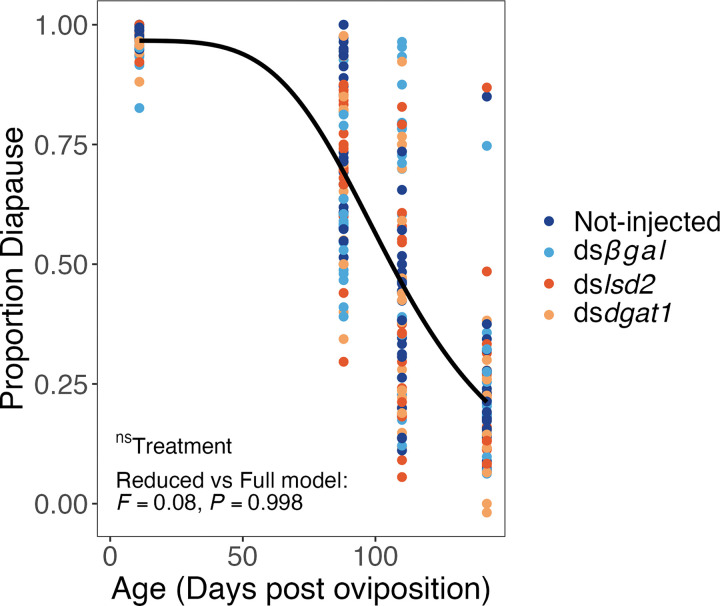
The proportion of eggs in diapause decreases over time. The black line represents the fitted three-parameter dose-response curve, which does not include treatment as a factor. Comparison of the reduced three-parameter model (excluding treatment) versus the full four-parameter model (including treatment) shows no significant effect of RNAi treatment on diapause termination timing (*F*-test results shown on figure).

The average proportion of eggs that remained in diapause while maintained at a constant 21°C, decreased from 0 to 142 days across all treatments and ranged from 70% at early diapause (~ 88 days post oviposition) to 45% at mid-diapause (~ 110 days post oviposition), and then to 22% at late-diapause (142 days post oviposition) (Fig. 6). There was no substantial difference between the 4-parameter and 3-parameter log-logistic models (ANOVA, *P* = 0.202), indicating that the effect of RNAi-treatment was not statistically significant.

### Overwinter Survival

**Figure 7:**
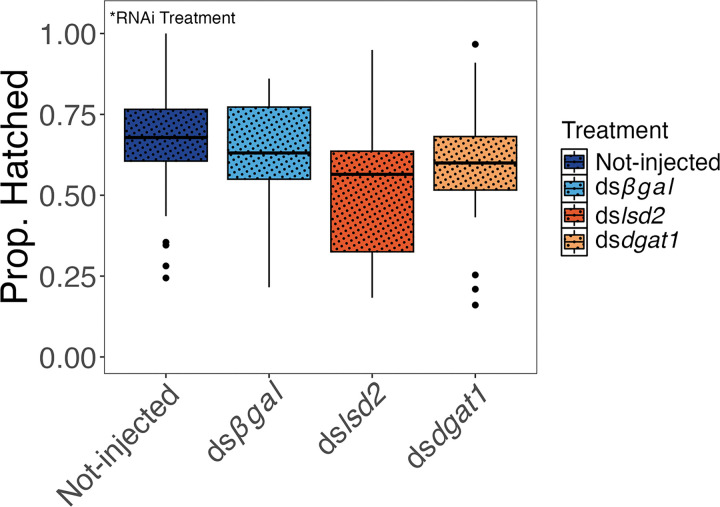
RNAi treatment affects overwinter survival of eggs hatched following a simulated winter. Box plots show the distribution of hatch rates of short-day eggs following different maternal RNAi treatments. Symbols and conventions as in Fig. 3.Statistical results comparing the full GLM model (including injection treatment) to the reduced model (excluding injection treatment) are shown. Despite a significant main effect of RNAi treatment, Tukey HSD post-hoc tests revealed no pair-wise differences.

RNAi treatment had a statistically significant effect on the proportion of eggs that hatched following a simulated winter (*F*_3,106_ = 2.99, *P* = 0.03) (Fig. 7). Pairwise comparisons revealed that eggs from ds*lsd2* injected mothers had marginally lower survival compared to eggs from non-injected mothers (*z* = −2.55, *P* = 0.053), but no other pairwise comparisons between treatments showed significant differences (all *P*-values > 0.05).

## DISCUSSION

Photoperiodic diapause is a prevalent form of adaptive phenotypic plasticity that enables overwinter survival in a wide range of temperate insects [3, 4]. In species with a maternally controlled photoperiodic diapause response, the photoperiod (daylength) experienced by the mother controls the diapause status of her progeny. Despite the crucial adaptive significance of maternally-controlled diapause, the specific mechanism(s) by which the maternal generation signals the offspring generation to enter diapause remain largely unresolved. Since the accumulation and storage of nutrients, particularly lipids, is a hallmark of diapause preparation and diapause maintenance [14, 16, 17, 37], we hypothesized that increased nutrient accumulation and provisioning mediate transgenerational diapause signaling in the vector mosquito, *Ae. albopictus*. The temperate population of *Ae. albopictus* used in this study provides an ideal system for testing this hypothesis, as it exhibits a well-characterized photoperiodic diapause response [38], with defined molecular processes [25, 30, 31], established metabolic [28] and hormonal signatures [27], and experimentally tractable maternal control of diapause initiation [23, 39]. To test this hypothesis, we used RNA-interference (RNAi) to suppress lipid metabolism transcripts in the maternal generation of *Ae. albopictus* and determine the impact on transgenerational lipid provisioning and both diapause and non-diapause traits.

### Triglyceride Provisioning and Larval Starvation Tolerance

RNAi targeting *lsd2* and *dgat1* in blood-fed females led to a 40–60% reduction in transcript abundance of each gene relative to RNAi targeting *βgal* (negative control) (Fig. 1a–d). This knockdown effect was reproducible under both long and short photoperiods. However, neither the triglyceride levels (Fig. 2) nor the starvation tolerance (Fig. 3) of 1st instar larvae were reduced in eggs produced by ds*dgat1*-injected females, indicating the energetic reserves of these eggs were not impacted by *dgat1* knockdown. This was a somewhat surprising result because the *midway* (*mdy*) gene in *D. melanogaster* encodes a DGAT enzyme [36] that shows strong similarity to the *Ae. albopictus* DGAT protein targeted here (68.17% identity, 84% query coverage). Expression of *mdy* is enriched in *D. melanogaster* nurse cells and loss of *mdy* expression leads to a reduction in neutral lipids in nurse cells. A likely explanation for the lack of a transgenerational effect of *dgat1* knockdown in the current study is that the triglycerides that were provisioned to the developing ovaries were synthesized in newly eclosed mosquitoes prior to blood feeding. Consistent with this interpretation, Ziegler and Ibrahim [40] report that 80% of egg lipids in *Ae. aegypti* are derived from fat body reserves that were established before consuming a blood meal, likely from nutrient carryover from the larval stage or from sugar sources [40]. Therefore, the transcriptional activity of *dgat1* following a blood meal may not impact the concurrent gonotrophic cycle. Rather, those transcripts might be used for the synthesis of triglycerides required for non-reproductive processes or for the development of secondary and tertiary oocytes in subsequent gonotrophic cycles.

In contrast to RNAi against *dgat1*, maternal RNAi against *lsd2* reduced both egg triglyceride levels and the starvation tolerance of 1st instar larvae (Figs. 2 and 3). LSD2 localizes to the surface of lipid droplets where it prevents catabolism by lipases and promotes lipid storage [32, 33]. Lipid droplets store neutral lipids, primarily in the form of triglycerides and cholesterol esters, which provide substrates for membrane synthesis and repair, as well as for hormones, secondary messengers and ATP generation [41]. The reduction in egg triglycerides following *lsd2* knockdown is consistent with the role of LSD2 in regulating lipid droplets and preventing lipid catabolism. Additionally, *lsd2* is highly expressed in the female germline of *D. melanogaster* during oogenesis, as well as in early syncytial blastoderm-staged embryos, and *lsd2* mutant *D. melanogaster* produce embryos with fewer neutral lipids [32, 35]. Since we did not measure *lsd2* mRNA abundance in early *Ae. albopictus* embryos, we did not determine whether RNAi against *lsd2* had an effect on maternal provisioning of transcripts during oogenesis and embryogenesis, or a direct effect on the packaging of triglycerides in oocytes (or a combination of both). Nevertheless, the significant reduction in triglycerides in eggs that were assayed 11 days after oviposition establishes a transgenerational effect of maternal *lsd2* transcript levels on embryonic lipid accumulation. Furthermore, when maintained in sterile water, 1st instar larvae produced by ds*lsd2*-injected mothers die on average one day earlier than 1st instar larvae produced by mothers of any other RNAi-treatment (ds*dgat1*, ds*βgal*, non-injected; Fig. 3). Since the starved 1st instar larvae did not have an opportunity to eat, the results thus demonstrate that a reduction in maternally provisioned energetic reserves carried over from the egg stage impacts offspring physiology.

The average quantity of triglycerides in eggs produced by short-day mothers was 1.88-fold higher than in eggs produced by long-day mothers (averaged across all treatments) (Fig. 2). This is consistent with previous studies in a wide range of insects[14, 42] including in *Ae. albopictus*, demonstrating that diapause eggs have greater overall lipid abundance, higher quantities of diglycerides and triglycerides, and altered metabolite profiles indicative of increased lipid storage and decreased lipid catabolism [28, 30]. Furthermore, the eggs produced by short-day ds*lsd2* injected mothers had similar quantities of triglycerides to non-diapause, long-day eggs produced by non-injected and ds*βgal* injected control mothers (Fig. 2). This provides a strong and experimentally direct basis to evaluate the specific role of triglyceride provisioning on transgenerational diapause signaling without introducing additional environmental variables or dietary changes that might have confounding effects on nutrient accumulation [14].

### Diapause Incidence

We hypothesized that lipids regulate the developmental trajectory of diapause vs. non-diapause embryos through a combination of increased maternal lipid provisioning and an offspring (embryo) nutrient-sensing mechanism. The rationale for this hypothesis is that lipid levels are often higher under diapause conditions [14–16], including in *Ae. albopictus* eggs [28, 30]. Further, since triglycerides are the primary energetic lipid used by insects [43], the quantity of stored triglyceride could indicate whether an individual has enough energy to survive throughout diapause, potentially serving as a signal for diapause initiation. Therefore, we predicted that short-day eggs with reduced triglyceride levels would avert diapause. However, ~ 97% of eggs produced by *lsd2* knockdown mothers reared under short-day conditions entered diapause (Fig. 4), even though they had similar quantities of triglycerides to long-day eggs produced by the negative control animals (Fig. 2). Diapause incidence was tightly distributed between an average 94–98% across all treatments, which is within the range of reported diapause incidences from previous studies using lab colonies collected from the same location reared under the same conditions [24, 27, 28, 44]. Therefore, these results do not support the hypothesis that short-day eggs with reduced triglyceride levels avert diapause.

The importance of lipids during insect diapause is well appreciated, yet the role of lipid levels as signals for diapause initiations remains unclear, particularly in a transgenerational manner. For example, knockdown of *fatty acid synthase 2 (fas2)* in adult cabbage beetles, *Colaphellus bowringi*, suppressed lipid accumulation but had no impact on the timing of diapause preparation or diapause incidence in adults [21]. Similarly, knockdown of the lipid metabolism genes, *lsd2, ACCase, FAS1, FABP, and FATP* in adults of the Colorado potato beetle, *Leptinotarsa decemlineata*, reduced triglyceride accumulation, but did not inhibit diapause initiation [22]. However, knockdown of *FAS1* and *ACCase* led to a prolonged diapause preparatory phase, and knockdown of *FADP* and *FATP* led to a shortened preparatory phase [22]. Interestingly, *ACCase and FAS1* are both involved in *de novo* fatty acid synthesis, whereas *FADP* and *FATP* are required for cellular localization. Thus, the prolonged diapause preparatory phase in the Colorado potato beetle following *FAS1* and *ACCase* knockdown suggests fatty acids and other lipogenesis intermediates, rather than triglycerides, may serve as alternative signals for the timing of entry into diapause, though they have yet to be tested in the context of transgenerational diapause signaling. Taken together, our results as well as these other functional studies, indicate that triglyceride and fatty acid levels are not sufficient to avert diapause initiation.

An alternate hypothesis regarding the role of nutrients as a signal for diapause is that poor nutritional conditions will induce diapause. In fact, previous studies have found that low protein diets and poor host quality increase diapause propensity in several species, including *Ae. albopictus* [45–51]. However, in our study, only, 3–7% of 11-day-old viable (embryonated) eggs remained unhatched (potentially in diapause), after two hatching stimuli were imposed (Fig. 4). These levels are also within the range of previously reported diapause incidences under long-day conditions for this population of *Ae. albopcitus* [24, 27, 28, 44]. It is important to note that the lack of hatching response by these eggs could be due to mortality that occurred subsequent to the completion of embryonic development rather than entry into diapause. Nevertheless, even under the conservative assumption that these unhatched eggs from LD mothers are in diapause, the results provide no support for the hypothesis that low rather than high triglyceride content induces diapause.

### Desiccation Resistance

Eggs produced by *lsd2* knockdown mothers were more prone to desiccation than all other injection treatments under both long-day and short-day photoperiod conditions (Fig. 5). These results suggest that *lsd2* is important for the storage of structural eggshell lipids as well as energetic lipids. The abundance and composition of structural lipids are associated with desiccation resistance and diapause in a range of insects [21, 52–54], including *Ae. albopictus* [29]. Structural lipids on the surface of insect eggs enhance the structural integrity of the egg and prevent water loss by forming protective cuticular hydrocarbons as part of the chorion [55, 56]. Notably, diapause *Ae. albopictus* eggs have more surface hydrocarbons and lower rates of water loss than non-diapause eggs [29]. This is likely related to increased abundance of *fatty acyl-coA elongase* transcripts in mature oocytes of short-day females, a gene that is associated with *de novo* hydrocarbon synthesis [29, 57]. Although lipid droplets are typically associated with triglyceride storage, they can also store long-chain fatty acid (LCFA) precursors, thereby impacting the availability of surface hydrocarbons for incorporation into the chorion [54, 58]. Thus, knockdown of *lsd2* likely prevents the accumulation and availability of surface hydrocarbons to the cuticle layer, rendering eggs more vulnerable to desiccation [33]. Increased desiccation resistance in diapause *Ae. albopictus* eggs not only improves survivorship under harsh winter conditions, but it also enables eggs to survive inadvertent, long-distance shipping transport, an important factor contributing to the global spread of the species [59]. Therefore, the loss of desiccation resistance following *lsd2* knockdown represents the loss of a key ecological adaptation that is crucial for diapause, as well as the invasion success of *Ae. albopictus* [60, 61].

### Diapause Termination

In addition to determining the role of lipids as a signal mediating diapause initiation, we also hypothesized that nutrient depletion would impact the timing of diapause termination. Past studies have shown that nutrient reserves of diapausing insects decline over the course of the winter [62]. Furthermore, in some species, larger individuals, which are thought to have greater nutrient stores than smaller individuals, will remain in diapause for longer durations [16, 17, 63]. However, recent work has revealed contrary patterns: flesh flies (*Sarcophaga crassipalpis*) that fed longer and were larger, terminated pupal diapause earlier than sparsely fed, smaller flies [14]. Thus, the role of nutrient status on the timing of diapause termination may vary among species and generalizations are not possible based on current data. Assuming triglyceride hydrolysis occurs at the same rate across RNAi treatments, we predicted that eggs entering diapause with lower quantities of triglycerides would deplete their reserves and terminate diapause earlier. However, we found no impact of RNAi treatment on the timing of diapause termination at any of the three timepoints that we tested (Fig. 6). This is similar to our finding that triglyceride content did not alter diapause initiation, underscoring the conclusion that triglyceride levels alone are not sufficient to cause either diapause initiation or termination in *Ae. albopictus*. The large variation in the timing of diapause termination, especially for the first two post-oviposition timepoints of 88 and 110 days (Fig. 6), may represent a bet hedging strategy with respect to the optimal seasonal timing of diapause termination. This interpretation has been proposed in other taxa, including weevils, *Curculio elephas* (larval diapause) [64] and gall midge *Hasegawaia sasacola* (prepupal diapause) [65].

### Over-winter Survival and Post-diapause Performance

Since lipid accumulation is a key element of the diapause phenotype, we predicted that lower triglyceride provisioning (as seen following ds*lsd2* treatment) would increase overwinter mortality. Our results demonstrate that eggs from ds*lsd2* injected adult females exhibited a marginally non-significant (*P* = 0.053) decrease in overwinter survival relative to eggs from non-injected females (Fig. 7), and a significant decrease in post-diapause larval starvation tolerance (Fig. 3).

Given the importance of lipid accumulation and storage during diapause across diverse insect taxa [3], it is surprising that overwinter survival was only marginally impacted by *lsd2* RNAi, especially since egg triglyceride levels were reduced to quantities similar to non-diapause eggs, and non-diapause eggs have significantly higher overwinter mortality rates than diapause eggs [66]. However, since technical problems prevented precise programming of diurnal temperature fluctuations for the duration of the simulated winter (see Methods, Overwinter Survival and Post Diapause Performance), it is possible that the conditions we used were not an accurate reflection of the energetic challenges of winter field environments. Nevertheless, the starvation-tolerance of 1st -instar larvae that hatched from overwintered eggs after maternal *lsd2* injection (Fig. 3) was 1.2 days shorter than the starvation-tolerance of 1st -instar larvae that hatched from non-overwintered eggs 11-days post-oviposition after maternal *lsd2* injection (Fig. 3), a pattern seen across all treatments. This reduced physiological performance after overwintering indicates that survival of the simulated winter in diapause did deplete energetic reserves. Taken together, the marginal decrease in overwinter survivorship in response to *lsd2* injection (Fig. 7), and the reduced larval starvation tolerance after over wintering (Fig. 3), provides important direct experimental support for the common inference that triglyceride levels contribute to overwinter survival and post-diapause performance in diapausing insects.

## Conclusion

Maternally-mediated embryonic diapause is a crucial form of transgenerational adaptive phenotypic plasticity in many temperate insects. We show that maternal expression of *lsd2* following a blood meal is important for transgenerational triglyceride provisioning to embryos during the first gonadotrophic cycle, but that maternal expression of *dgat1* is not. Despite a significant reduction in egg triglycerides following *lsd2* RNAi, neither diapause initiation nor diapause termination was affected by *lsd2* RNAi. It is likely that nutritional signaling interacts with multiple factors that have been implicated during diapause, including hormonal signaling [10], circadian regulation[67, 68] and epigenetic modification[69] to regulate diapause initiation and termination. However, eggs from mothers injected with ds*lsd2* exhibited a decrease in desiccation resistance, a diapause-associated phenotype, and post-diapause larval starvation tolerance. These results imply that altered diapause energetics, which may be associated with changing ecological conditions such as an increased prevalence of winter heat waves, will impact the fitness of overwintering insects. Overall, these results support the important role of maternal lipid provisioning to diapause-associated fitness, but not as a transgenerational signal regulating diapause initiation or termination.

## METHODS

### Mosquito Rearing

A lab population of *Ae. albopictus* was established with pupae and larvae collected from an auto-salvage yard in Manassas, Virginia in 2018. Animals were reared under standard laboratory conditions in a walk-in, controlled-temperature (CT) room at 21°C, 80% relative humidity, 16h light:8h dark for 16 subsequent generations [24]. Briefly, eggs were stimulated to hatch in a 5.5L container with 2L of deionized water and 1 mL of a larval food slurry prepared by combining 1L deionized water, 120g dog-food (Nutro Ultra Small Breed Puppy, Nutro Products Inc., Franklin, TN, USA) and 40g frozen brine shrimp ( Sally’s Frozen Brine Shrimp, San Francisco Bay Brand, Newark, CA, USA) [70]. Larvae were reared in 5.5L bins at a density of 200 larvae per 2L deionized water. Every Monday-Wednesday-Friday (M-W-F), larvae were transferred to clean bins with fresh deionized water and fed 1 mL of larval food slurry. Pupae were transferred into 2L adult cages and provisioned with organic raisins (Newman’s Own, Westport, CT, USA) to allow *ad libitum* sugar feeding. Beginning one week after the last pupae were collected, females were allowed to blood feed on a human host. Georgetown University Institutional Review Board has determined that mosquito blood feeding is not human research and does not require IRB approval; however, the blood feeding protocol has been approved by the Georgetown University Office of Health and Safety (OHS) and performed in accordance with the relevant guidelines and regulations outlined by the OHS. Informed consent was obtained from all individuals involved in blood feeding. Beginning three to four days after blood feeding, a dark oviposition cup lined with unbleached paper towel and half filled with DI water was placed in each cage for egg collection. Egg papers were collected and replaced every two to three days. Collected egg papers were left moist in individual petri dishes for two to three days and then gently air dried and stored within a sealed Tupperware container containing a small cup of water to maintain local humidity.

For the experimental generation, F16 eggs were hatched and larvae were reared as described above. Pupae were collected daily and were either maintained under unambiguous long-day (LD), non-diapause inducing conditions (16h light:8h dark, 21°C, 80% relative humidity) in the CT room, or under unambiguous short-day (SD), diapause-inducing conditions (8h light:16h dark) in photoperiod cabinets housed within the CT room as described previously [24, 39]. Male and female pupae were sorted and transferred into separate 2L adult cages. Female cages were checked daily and non-eclosed female pupae were moved into new cages such that each female cage contained individuals that had all eclosed within 24 hours. All cages were provisioned with organic raisins (Newman’s Own, Westport, CT, USA) to allow *ad libitum* sugar feeding. Six to seven days after pupation, raisins were removed from the female cages for 24 hours, and females were allowed to blood feed on a human host on the day of dsRNA injection (described below).

### dsRNA Synthesis for RNAi injection

Double-stranded RNA synthesis was performed as described previously [71]. Briefly, ten *Ae. albopictus* females from the Manassas, Virginia colony described above were reared as described above and flash frozen in liquid nitrogen 72 hours after blood-feeding. Individuals were submerged into RNA*later*^™^ (Invitrogen, Waltham, MA, USA) and all head, wing and leg tissue was discarded. Any undigested blood bolus was dissected from the abdomen, while the remaining thorax and abdomen were pooled into a single sample. The tissue was then homogenized in 2mL TRIzol^™^ (Invitrogen, Waltham, MA, USA). One mL of the homogenate was retained for subsequent phenol-chloroform RNA extraction, followed by an isopropanol precipitation according to the manufacturer’s directions. One microgram of RNA was converted to cDNA using BioRad’s iScript^™^ cDNA synthesis kit (Bio-Rad, Hercules, CA, USA). cDNA was used as template to amplify a 490 bp region of *lipid storage droplet protein 2* (*lsd2)* (XM_029868889.2) and a 377 bp region of *diacyl glycerol-o-acetyl transferase 1 (dgat1)* (XM_019692235.3). A 189 bp region of *Beta galactosidase (βgal*) (negative control gene) was amplified from plasmid DNA. dsRNA was synthesized using the T7 RiboMAX Express RNAi System (Promega, Madison, WI, USA), according to the manufacturer’s instructions and purified through a Microcon^®^ DNA Fast Flow filtration device (MilliporeSigma, Burlington, MA, USA). See Supplementary Table S1 for primers used to produce both cDNA template and dsRNA.

### PCR Cycling for dsRNA synthesis

To produce template for dsRNA synthesis, an initial PCR reaction was performed to amplify a 490 bp region of *lsd2* and a 377 bp region of *dgat1.* The initial PCR reactions for each gene were performed in a total volume of 25μL containing 2μL cDNA, 12.5μL FailSafe PCR 2X PreMix E (LGC Biosearch Technologies, Hoddeson, UK), 0.37μL GoTaq^®^ DNA Polymerase (Promega, Madison, WI, USA), and 5pmol of forward and reverse primer (Supplementary Table S1) under the following cycling conditions: 95°C for 5 minutes followed by 35 cycles of denaturation at 95°C for 30 seconds, annealing at 60.4°C for 30 seconds, extension at 72°C for 1 minute, and a single final extension cycle at 72°C for 5 minutes. PCR bands were gel excised and purified using a QIAquick Gel Extraction Kit (QIAGEN, Germantown, MD, USA). The purified PCR products for each gene were then reamplified in two separate reactions to produce template with the T7 promoter on both strands. One reaction used the forward primer with the T7 promoter added to the 5-prime end and the reverse primer, while the other reaction used the reverse primer with the T7 promoter added to the 5-prime end and the forward primer (Supplementary Table S1). Additionally, a 189 bp region of *βgal* was amplified from a strain of *Escherichia coli* DH5α(pCL-lacZ) in two separate reactions to produce template with the T7 promoter on both strands. PCR reactions were performed in a total volume of 50μL containing 2μL purified PCR product, 25μL FailSafe PCR 2X PreMix E (LGC Biosearch Technologies, Hoddeson, UK), 0.74μL GoTaq^®^ DNA Polymerase (Promega, Madison, WI, USA), and 10pmol of forward and reverse primer under the following cycling conditions: 95°C for 5 minutes followed by an initial 15 cycles of denaturation at 95°C for 30 seconds, annealing at 60°C for 30 seconds, extension at 72°C for 1 minutes, then an additional 25 cycles of denaturation at 95°C for 30 seconds, annealing at 65°C for 30 seconds, extension at 72°C for 1 minutes. PCR bands were purified using a QIAquick PCR Purification Kit (QIAGEN, Germantown, MD, USA).

### RNAi Injections

dsRNA Injections were performed as described previously [71]. Seven to 8 day old adult females (age post-pupation) were blood fed at ZT 0–2 on the day of dsRNA injection. Beginning two hours after blood feeding, females were lightly anesthetized with CO_2_ for 10 seconds and placed on a petri dish on ice for 10–20 minutes. While on ice, fully engorged females were intrathoracically injected with 0.138μL of 7.25ng/μL dsRNA (total 1ug dsRNA) targeting either *lsd2, dgat1* or *βgal* (to control for dsRNA injection) using a Nanoject III (Drummond Scientific, Broomall, Pennsylvania, USA). A subset of non-injected mosquitoes was also anesthetized and placed on ice for the same duration of time as injected mosquitoes to serve as a negative control. Replicate cages were established for each injection treatment (ds*lsd2*, ds*dgat1*, ds*βgal*) to perform qRT-PCR assessment of RNAi efficiency, and replicate cages for non-injected as well as all injection treatments were established to collect eggs for triglyceride quantification, diapause incidence and post-diapause performance assays (described below). Once replicate cages were established for each treatment, dead mosquitoes were removed from cages up to four days post blood feeding to evaluate survivorship. See Supplementary Table S2 for details on the number of replicate cages and individuals used in each assay.

### qRT-PCR to Assess RNAi Efficiency

dsRNA injected females (ds*lsd2*, ds*dgat1 or* ds*βgal*) (see above) were placed into separate 2.5-quart plastic cages with organic raisins. Males were also added to each cage at a ratio of 2 males for every 5. Injected females were flash frozen in liquid nitrogen 72 hours post-blood meal and stored at −80°C. Total RNA was extracted from pools of thorax and abdomen as described above in the dsRNA synthesis section and used to determine relative transcript levels by qRT-PCR. Primer sequences for qRT-PCR amplification of the reference gene ribosomal protein S6 (*AalRpS6*, accession number: AF154066), *lsd2*, and *dgat1* are described in Supplementary Table S1. All primers produced a single melt curve and standard curves were generated to verify each primer met the MIQE guidelines (Supplementary Table S1).

qRT-PCR was performed in 96-well plates using a CFX96 Touch^™^ Real-Time PCR Detection System (Bio-Rad, Hercules, CA, USA) and with Agilent’s Brilliant II SYBR^®^ Green QRT-PCR 1-Step Master Mix (Agilent, Santa Clara, CA, USA). All qPCR reactions were performed in triplicate in a total volume of 12.5μL with 25ng RNA and 2.5pmol primers using the following cycling parameters: 95°C for 3 minutes followed by 45 cycles of 95°C for 10 seconds, 58°C for 30 seconds and 72°C for 30 seconds. Melt curve analysis of PCR products verified only one product was amplified in each reaction.

The 2^−ΔΔCt^ method [72] was used to determine the relative mRNA abundance (i.e., fold change) of *lsd2* and *dgat1.* First, the average CT of *RpS6* was subtracted from the average CT of *lsd2* and *dgat1* (separately) for each sample to determine the normalized abundance of *lsd2* and *dgat1* in each sample. Next, the normalized abundance of target gene for each sample was subtracted from the average normalized abundance of the target gene in ds*βgal* mosquitoes. This value was used to exponentiate the base – 2 to calculate the relative fold change in expression of each target gene.

### Egg Collections for Triglyceride Quantification

To collect eggs for triglyceride quantification, ds*lsd2*, ds*dgat1*, ds*βgal* or non-injected females were placed into separate 2.5-quart plastic cages. In total, six replicate cages per treatment per photoperiod were established with between 29–42 females per cage (Supplementary Table S2). Males were added to each cage at a ratio of 2 males for every 5 females. Beginning four days after blood feeding, a dark oviposition cup lined with unbleached paper towel and half-filled with DI water was placed in each cage for egg collection. Egg papers were collected and replaced daily for up to 11 days after blood feeding. Collected egg papers were left moist in individual Petri dishes for 48–72 hours and then gently air dried and stored within a sealed Tupperware container containing a small cup of water to maintain local humidity. Eleven days after collection, intact eggs were counted (flattened or collapsed eggs were removed), gently brushed into individual microcentrifuge tubes, flash frozen in liquid nitrogen, and stored at −80°C.

### Total Lipid Extraction and Triglyceride Quantification

Between 350–480 eggs were pooled across collection days for each of the six biological replicate cages per treatment. Eggs were homogenized in chloroform/methanol (1:1) in 2 mL glass homogenizers and each homogenate was transferred to 1.5 mL microcentrifuge tubes sealed with parafilm. Tubes were sonicated for 30 min, centrifuged at 13k rpm for 5 min, and the supernatant (containing total lipids) was transferred to individual 1.85 mL borosilicate vials (VWR^®^, West Chester, PA). Vials were heated to 90°C to evaporate the solvent and retain total lipids.

The total lipid in each vial was then resuspended in 200 μL chloroform and sonicated for 5 min. Twenty-five microliters of the resuspension was transferred to three separate technical replicate 1.85 mL borosilicate vials. Vials were heated again to 90°C to evaporate the chloroform and 200 μL of Infinity Triglyceride Reagent (ThermoFisher Scientific, Waltham MA, USA) was added to each vial after cooling to room temperature (~ 20 minutes). Vials were sonicated for 8 min and 180 μL of the sonicated reagent was transferred to a single well of a clear, round bottom, polystyrene, 96 well plate (Corning Inc, Corning NY). Additionally, a triglyceride standard was prepared as follows: 10 mg of corn oil was suspended in 10 mL of chloroform and diluted 1:10 to make a 0.1 mg/mL stock. Twelve aliquots each of 25, 50, 100, 200, 300 and 400 μL of the 0.1 mg/mL stock were transferred into fresh 1.85 mL borosilicate vials and heated at 90°C to evaporate the chloroform. Standards were stored at −20°C prior to use. For each plate, 200 μL of Infinity Triglyceride Reagent was added to triplicate vials of each standard concentration. Vials were sonicated for 8 min and 180 μL of the sonicated reagent was transferred to a single well of a 96 well plate (Corning Inc, Corning NY). For each photoperiod treatment (SD, LD), two plates were used to determine TG quantities; each plate contained three biological replicates per injection treatment run in triplicate. This resulted in 2 plates per photoperiod (four individual plates total). Absorbance at 500 nm was read using a microplate reader (BioTek SynergyH1). A linear curve was generated by averaging absorbance values and known concentrations of lipid standards to calculate the lipid content of each biological replicate.

### Starvation Tolerance

To assess the impact of maternally provisioned energetic reserves on post-egg hatch larval performance, larval starvation tolerance was measured as described previously in Batz et al. (2020) [38]. This assay measures the time to death for freshly hatched larvae maintained in sterile water as a proxy for energetic/metabolic reserves at the time of hatching. Long-day eggs produced by mothers that were either dsRNA injected or non-injected were collected from 8 replicate cages per treatment (as described above), and 11 days after oviposition, eggs were stimulated to hatch in 90mm × 15mm Petri dishes filled with 20 mL DI water and three drops of dog-food slurry. Plates were checked for hatch every 3.5 hours and hatched larvae were placed into a new plate with fresh DI water. Larvae were transferred into three additional plates with fresh DI water to remove residual food before being placed into single wells of sterile 24-well tissue culture plates (Corning Inc, Corning NY) containing 1mL of autoclaved DI water. Wells were examined daily for mortality by probing larvae with a sterile pipette to check for movement, and days to death were recorded for each larva. The rearing details for larvae that hatched from eggs produced by SD mothers following a simulated winter are described below (see Overwinter Survival, Post Diapause Starvation Tolerance).

### Diapause Incidence

To determine the effect of RNAi treatment on diapause incidence, eggs from 8 LD and 11–12 SD cages of each injection (and non-injection) treatment were collected, dried and counted as described above. Eleven days after oviposition, eggs were stimulated to hatch in 90mm x 15mm petri dishes filled with 20 mL DI water and three drops of dog-food slurry. Stimulated eggs were maintained at their respective photoperiods for three days at 21°C. After three days of stimulation, the number of larvae was counted, and egg papers were gently air dried and stored in petri dishes within a sealed Tupperware container containing a small cup of water to maintain local humidity. One week after counting the first hatch, egg papers were restimulated to hatch for three days and larvae were counted as described previously. Unhatched eggs were then submerged in a bleach solution overnight to clear the pigment in the chorion of the unhatched eggs [73]. Eggs were examined under a stereomicroscope to identify fully-developed pharate larvae, which were considered to be in diapause. Diapause incidence (DI) was calculated as DI = (number of embryonated unhatched eggs)/(total number of hatched larvae + embryonated unhatched eggs) [24, 25, 38, 74] .

### Desiccation Resistance

To determine the effect of RNAi treatment on desiccation resistance, eggs from 6 LD and 9 SD cages of each injection (and non-injection) treatment were collected and dried as described above. Eleven days after oviposition, the total number of eggs on each oviposition paper were counted under a Zeiss Stemi-2000c Stereo Microscope. Desiccated eggs (those that were collapsed or flattened) were also counted and removed. The proportion of eggs that were desiccated was calculated as # collapsed eggs/total eggs.

### Diapause Duration

To determine the effect of RNAi treatment on the timing of diapause termination, eggs from 16–25 SD cages of each injection (and non-injection) treatment were collected and dried. In this experiment, eggs were collected every three days in order to increase the number of eggs per oviposition paper. Eggs from each treatment were collected from 16–25 replicate SD cages per treatment and maintained at 21°C in in petri dishes within SD photoperiod cabinets. Two to five days after oviposition, eggs were gently air dried and stored in sealed Tupperware containers containing a small cup of water to maintain local humidity. Desiccated eggs were removed from egg papers 11–14 days post-oviposition. Eggs were removed from the SD photoperiod cabinet and transferred to a LD photoperiod 63–66 days post-oviposition. Eggs were stimulated to hatch during early diapause (85–88 days post-oviposition; ~12 weeks), mid diapause (107–110 days post-oviposition; ~15 weeks), and late diapause (139–142 days post-oviposition; ~20 weeks) in ~ 15 mL DI water with one drop of larval food slurry in 60 × 15mm petri dishes. Eggs from one to two independent egg papers were stimulated for each of the three termination date ranges. Larvae were counted 3 days after hatching, and egg papers were gently air-dried and stored in the CT room for 5 days before re-stimulating to hatch as above; any additional larvae that hatched were counted 3 days after the second hatch. Diapause duration was measured by quantifying the reduction in diapause incidence over time after 85 to 142 days and calculated as (number of hatched larvae)/(total number of stimulated eggs).

### Overwinter Survival and Post Diapause Performance

To assess overwinter survival and post-diapause performance, eggs were collected from 26–30 replicate SD cages and dried as described in the diapause termination section. After 18–21 days at 21°C, the eggs were exposed to simulated winter conditions in a Percival I-36VL incubator (Percival Scientific, Perry, IA). Winter conditions were simulated by setting the incubator temperature control to sinusoidally oscillate each day between 9°C and 20°C. However, five days into the overwinter treatment, the thermostat malfunctioned and the average oscillation temperature decreased to 5.6°C below the set point. The incubator malfunctioned a second time 25 days into the overwinter treatment when the temperature dropped to −12.8°C, rose to 7.9°C, and dropped back down to −13.2°C over the course of seven hours. At this point eggs were transferred to a different Percival I-36VL incubator (Percival Scientific, Perry, IA) set to constant 10°C (SD photoperiod) for the remainder of the overwinter treatment.

Between 170–173 days post oviposition, eggs were moved from the overwinter treatment (SD, 10°C) to LD conditions at 21°C. After one week, the eggs were stimulated to hatch in 90mm x 15mm petri dishes filled with 20 mL DI water and three drops of dog-food slurry. Three days after hatching, egg papers were removed, rinsed with DI water, re-dried and stored for one additional week before being restimulated to hatch. Hatched larvae were counted three days after the first and second stimulation and the percent survival was calculated as (total number hatched larvae)/(total number eggs stimulated to hatch). On the first day eggs were stimulated to hatch, petri dishes were checked every 3.5 hours to collect fresh larvae to set up post diapause larval starvation assays (details on larval starvation assay described above).

### Statistical Analyses

To determine the effect of double-stranded RNA injection targeting *lsd2* and *dgat1*, a two-sample *t*-test was used to compare the transcript levels of the target gene relative to ds*βgal* injected females under SD conditions. Because LD samples were not normally distributed, a Wilcoxon rank sum test was used to compare the transcript levels of the target genes relative to ds*βgal* injected females.

To determine the effects of RNAi treatment and photoperiod on egg triglyceride content, starvation tolerance, diapause incidence, and desiccation resistance, we initially fit a two-way ANOVA model incorporating injection treatment, photoperiod, and their interaction. Model assumptions were assessed by examining residuals using Levene’s test for homogeneity of variance and the Shapiro-Wilk test for normality [75]. For triglyceride content, which met both assumptions, we proceeded with the standard two-way ANOVA. If a significant main effect was detected with no significant interaction, we reran the ANOVA without an interaction and performed Tukey’s Honest Significant Difference (HSD) test to identify differences between groups.

For response variables that violated parametric assumptions (starvation tolerance, diapause incidence, and desiccation resistance), we implemented generalized linear models (GLMs) with appropriate error distributions and link functions as described below. Days to starvation was analyzed using a Poisson GLM with a log link function. For proportion data (diapause incidence and egg desiccation), we incorporated egg counts in the model structure and used quasibinomial GLMs with logit links to account for overdispersion. The significance of fixed effects and their interactions was assessed through model comparisons. Specifically, for the Poisson model (days to starvation), we compared full models to reduced models using ANOVA with chi-square tests. For quasibinomial models (diapause incidence and egg desiccation), we used ANOVA with *F*-tests to account for overdispersion. Statistical significance was set at α = 0.05. For all GLMs, when a significant main effect and interaction was detected, we conducted pairwise comparisons of all treatment and photoperiod combinations using estimated marginal means (emmeans package; Lenth, 2024), and used the Tukey method to control for multiple comparisons. When significant main effects were detected without an interaction effect, we conducted pairwise comparisons on each significant main effect model without the interaction using estimated marginal means (emmeans package; Lenth, 2024), and Tukey’s method to control for multiple comparisons.

To determine the effect of RNAi treatment on diapause duration, we combined diapause termination data with SD diapause incidence data at 11 days post oviposition and fit data points with a dose response model where diapause incidence was the dependent variable and age (days post oviposition) was the dose variable. A four-parameter log-logistic model was used with lower and upper limits fixed at 0 and 1, respectively. The slope and inflection points were allowed to vary and estimated from the data, with the inflection point corresponding to the age at which 50% of eggs terminate diapause. Individual treatment cages were used as biological replicates and diapause incidence at each time point across replicates was averaged in order to construct a full dose-response model with treatment as a fixed factor. The four-parameter log-logistic model was compared to a reduced dose-response model excluding treatment as a factor via ANOVA to determine if RNAi treatment impacted the duration of diapause.

To determine the effect of RNAi treatment on overwinter survival, we used a quasibinomial GLM with logit links to account for overdispersion because the data violated assumptions of normality. We assessed the effect of treatment by comparing the full model (including injection treatment) with the reduced model (excluding injection treatment) using ANOVA with *F*-tests. Statistical significance was set at α = 0.05. We conducted post-hoc pairwise comparisons among all treatment groups using estimated marginal means (emmeans package; Lenth, 2024) and adjusting for multiple comparisons using the Tukey method. All statistical analyses were performed in R version 4.3.2 (R Core Team, 2023).

## Supplementary Material

Supplementary Files

This is a list of supplementary files associated with this preprint. Click to download.

• Heiligetal.SupplementalMaterialScientificReports.xlsx

## Data Availability

The datasets generated during and analyzed during the current study are available from the corresponding author on request.
